# The challenges of primary health care nurse leaders in the wake of New Health Care Reform in Norway

**DOI:** 10.1186/s12912-016-0187-x

**Published:** 2016-11-24

**Authors:** Wivi-Ann Tingvoll, Torill Sæterstrand, Leon Mendel McClusky

**Affiliations:** 1 Department of Health and Care, Faculty of Health Sciences, University of Tromsø - The Arctic University of Norway, Campus Narvik, Narvik, Norway; 2 Faculty of Professional Studies, Nord University, Bodø, Norway

**Keywords:** Nurse leaders, Community health care service

## Abstract

**Background:**

The local municipality, whose management style is largely inspired by the New Public Management (NPM) model, has administrative responsibilities for primary health care in Norway. Those responsible for health care at the local level often find themselves torn between their professional responsibilities and the municipality’s market-oriented funding system. The introduction of the new health care reform process known as the Coordination Reform in January 2012 prioritises primary health care while simultaneously promoting a more collaborative and multidisciplinary approach to health care. Nurse leaders experience constant cross-pressure in their roles as members of the municipal executive team, the execution of their professional and administrative duties, and the overall political aims of the new reform. The aim of this article is to illuminate some of the major challenges facing nurse leaders in charge of nursing homes and to draw attention to their professional concerns about the quality of nursing care with the introduction of the new reform and its implementation under NPM-inspired municipal executive leadership.

**Method:**

This study employs a qualitative design. In-depth interviews were conducted with 10 nurse leaders in 10 municipalities, with a phenomenological-hermeneutic approach used for data analysis and interpretation.

**Result:**

Findings highlighted the increasingly complex challenges facing nurse leaders operating in the context of the municipality’s hierarchical NPM management structure, while they are required to exercise collaborative professional interactions as per the guidelines of the new Coordination Reform. The interview findings were interpreted out of three sub-themes 1) importance of support for the nurse leader, 2) concerns about overall service quality, and 3) increased tasks unrelated to nursing leadership.

**Conclusion:**

The priorities of municipal senior management and the focus of the municipality’s care service need clarification in the light of this reform. The voices of those at the frontlines of the caring services need to be heard as the restructuring of the caring services may have implications both for funding allocation and for the quality of patient care.

**Electronic supplementary material:**

The online version of this article (doi:10.1186/s12912-016-0187-x) contains supplementary material, which is available to authorized users.

## Background

The Norwegian public service, like those of other developed countries, is largely governed in accordance with market-oriented principles and has adopted a flattened, two-level management structure inspired by the New Public Management (NPM) model [1–4]. Public health care in Norway is organised in two spheres, namely the primary health care services, managed mainly by nurse leaders in the employ of municipalities, and the hospital-based specialist services, administered by regional health authorities. Whereas the NPM model may be readily applied to the governing of other public services, the model’s suitability for the administration of nursing and health care that, in essence, entails a round-the-clock service for patients with widely varying and rapidly changing acute and chronic disease conditions, is less than ideal. Studies in several countries, including Australia, the UK and Switzerland, have drawn attention to the impact of NPM on the nursing profession [5–7]. For example, studies of NPM-inspired health care reform in Australia and Switzerland reported that nurse leaders were under growing pressure concomitant with an increasing complexity of the management of nursing services [7–9], leaving them with no time for critical reflection over the quality of medical care [6].

The introduction of new health care reform in 2012 in Norway, namely the Coordination Reform [10], added another level of complexity onto the largely NPM-inspired administration of health care. The reform involves judicial and organisational changes, which in essence means that the municipal health services and care services are allocated a greater share of the health care budget such that health care expenditure in the specialist health care services could be curtailed [11–13]. In formulating the new envisaged role for the municipalities, the reform states that “*municipalities should ensure that the patient receives the best effective health care through cohesive patient pathways ……….. and the needs of the patients should be identified as early as possible so that services can be called in*” [10]. The latter changes proposed by the new reform have consequences for nursing leadership, with stipulated responsibilities including preventative health care, identifying medical needs as early as possible, and mapping “cohesive” pathways for every patient under the care of his/her staff. Increased and closer collaboration with the specialist health care services and other health care professionals (e.g., occupational and physiotherapists) are implicit in the identification of medical needs and mapping of patient pathways. It becomes clear then that the execution of leadership responsibilities within the context of the flat two-level management model of NPM places additional administrative demands on the nurse leader [14–20]. Restructuring and adjustments will have budgetary consequences for the current management model as well, especially as regards the definition of goals and the outlining of resource needs (personnel, competence and financing [21–24]. Current infrastructure, resources and skills in the municipality can barely accommodate the proposed changes, all of which may affect health care quality standards and weaken the aims and intentions of the reform. With the overall reduction in specialist health care costs in the country another major aim of the reform, the latter therefore also proposes new guidelines for the reduction in hospital-bed occupancy rates and efficient discharge of patients into the municipal care services, while the frail and elderly comprise a large component of all hospital patients [1, 10, 21, 25]. These changes highlight the necessity for the development of clear guidelines for the definition of discharge-readiness of hospital patients, which is also a prerequisite for the proposed closer collaboration between the municipalities and hospitals [26]. Tasked with the overall managerial responsibility of admitting and administering care to the large body of elderly patients discharged from the hospitals, the nurse leaders managing the municipality’s nursing homes are key stakeholders and partners in the reconfigured health care landscape under the new Reform. This paper aims therefore to highlight the challenges and, at times, conflicting demands facing these nurse leaders, who also sit on the municipal executive team, since the introduction of the Coordination reform [10].

## Method

### The study field

The informants in this study were nurse leaders from 10 middle-sized municipalities, each serving a population of 5000–15000 inhabitants. In these municipalities, the structuring of the health services and care services followed a two-level organizational model, with the nursing homes and home care services organised as separate results units directly under the auspices of the municipal manager. The selection criteria for the informants, included that they had a minimum of 5 years’ managerial experience, had managerial responsibility for the nursing homes division in a municipality that served a minimum of 5000 inhabitants, and that they were stationed at the care facility they managed. All the sampled nurse leaders (aged 38 to 60 years) had postgraduate qualifications in leadership, all typical of the credentials and professional experience of the suitably qualified nurse leader in Norway. The municipalities were approached in writing for permission to get access to the informants.

### Data collection and analysis

This qualitative study employed the phenomenological-hermeneutic method [27], during which the data were collected by way of interviews of 1–1½ hours duration. The in-depth interviews, conducted jointly by the first and second authors in the offices of the informants, were based on a semi-structured questionnaire (Additional file 1) and other open in-depth questions that followed up on the information revealed by the informants about their experiences and challenges in the wake of the introduction of the Coordination Reform.

The interviews were recorded and the sound files subsequently transcribed word-for-word. The text files were returned to the informants for verification prior to commencing with processing of the data. The latter was carried out by the first and second authors, and followed Kvale et al.’s [27] three-step phenomenological-hermeneutic analysis of interviews, (i.e., (1) self-understanding, (2) common sense and (3) theoretical interpretation). Step one of Kvale et al.’s analysis summarized what the informants themselves understood from the interviews. In step two, the analysis involved a second probing dialogue between the researchers and the transcribed text, with any additional information noted down. The information that emanated from this translation process was integrated with the researchers’ prior academic knowledge and experience on the topic being researched (see [1, 26]), all while the researchers maintained their impartiality and distance from the experimental material. The third step involved the application of theory in the interpretation of the meanings of the statements and expressions of the informants using a phenomenological-hermeneutic approach.

### Research ethics

Ethical approval for the study was obtained from the Norwegian Social Science Data Services. The processing of the data and execution of the study was in accordance with the research ethics guidelines of the Helsinki Declaration. The informants received both oral and verbal information of the study, and were informed about their rights to withdraw from the study at any time without explanation, if they so wished. Sound files were deleted after transcription and verification of the transcribed material. Declaration of consent was sent back to the first author to safeguard the confidentiality of the informants.

## Results

The interview findings were broadly grouped as follows into three main themes and several sub-themes (see Table 1):

**Table 1:** Summary of findings

Theme	Sub-theme
Importance of support for the nurse leader	Increased need for qualified staff for an increasingly demanding 24-h care service. Lack of understanding from top leadership. Limited space for nursing care concerns. Demand for strict budgetary discipline.
Concerns about overall service quality	More hospital-discharged patients with increased treatment and care needs. Concerns about quality of treatment and care.
Increase in tasks unrelated to nursing leadership	Many stakeholders to relate to for nurse leaders. Constant re-prioritising of leader tasks.

1. Importance of support for the nurse leader
2. Concerns about overall service quality
3. Increase in tasks unrelated to nursing leadership

### Importance of support for the nurse leader

A recurring theme in all of the interviews was that this particular division of the public services sector, unlike the municipality’s other divisions (e.g. schools), operated with different time and personnel constraints, including shift work. Health care was a round-the-clock service and was reliant on the efforts of an appropriately trained and committed staff for its efficient functioning. The nurse leader managed a nursing staff who have to continuously admit a steady stream of discharged patients who are still in need of acute care interventions (e.g., pain relief and wound treatment) as the hospitals strove to reduce bed occupancy rates. Thus, some of the hospital care was still administered by the nurses of the municipality’s care service.

Several of these nurse leaders said:

*We in the deputy mayor group all work with the same items on the agenda. There are more overarching matters. There is little room to discuss the nursing and care services. The problem is that the others are not appropriately informed about a 24-h operation. They can school others and other stuff, but they know absolutely nothing about round-the-clock nursing!*

The working relationships among the municipality’s executive team members, which includes the nurse leader, appeared to be good, but the nurse leader thought that the other members of the executive team lacked any understanding of daily bedside nursing practice. Feelings of lonesomeness were prevalent among nurse leaders. One nurse leader even contacted the work guidance centre of another municipality for advice and guidance.

There was a clear need at middle management level for an arena to discuss difficult cases, as one informant also stated:

*I think it is useful to cooperate with colleagues… it is important that we are placed together and can support each other.*

These expressions were interpreted to mean that nurse leaders placed a premium on collegial support. Situations of conflicts arose rather quickly during times of organisational restructuring as staff operated under great pressure. Personnel conflicts were considered as especially demanding and consumed most of their time.

The nurse leaders felt that the support they received from other colleagues was important for them to continue in their job. One nurse leader stated:

*Pressure on the manager role is huge, and everyone is so busy. Then it is good to have colleagues also in the same manager position who are able to understand the situation.*

It seemed that the pressure was increasing at middle manager level. Although the Coordination Reform emphasised the importance of improved collaborative interaction, even among the municipality’s various divisions, the nurse leader still experienced a sense of loneliness professionally.

As a logical consequence of the municipalities’ NPM style of governance, the deputy mayor’s adherence to the budget did not resonate well with the nurse leaders, and several expressed themselves as follows:

*I feel that it has worked out well thus far. But I despair a bit because “it doesn’t matter what happens as long as it’s within the budget”. You are not noticed and don’t get support. One feels burned out.*

These frustrations were interpreted to indicate poor professional understanding and support from other members of the municipal executive team regarding the inadequate budgetary allocation for the care services as per the requirements of the Coordination Reform. It seemed that nurse leaders were not afforded the opportunity to put forward their professional arguments prior to the budget negotiations.

### Concerns about overall service quality

The municipalities surveyed in this study each had different ways of structuring the care service. Some of the nurse leaders were tasked with numerous responsibilities, including that for personnel, finances and professional responsibility, with the latter considered as the most burdensome. The number of nurse leader positions were even reduced in some municipalities, with responsibility and pressure concomitantly increased on the remaining nurse leaders. One described the situation as follows:

*The pressure down the management chain has gotten bigger and bigger. It is increasingly expected of one to also be a conflict solver. You need to know some law as there are more and more rules governing us. I have therefore started out in law studies because of this.*

These revelations testified of the increased amounts of time a nurse leader had to spend on personnel conflicts during this time of organisational restructuring, all often the outflow of the increasing pressure on staff to execute their duties as required by the new reform.

Another one expressed it this way:

*There is a lot of focus on finances and little on service quality. We do not have the possibilities to push through the developments in quality we so much wish to have. This is frustrating.*

This was interpreted to mean that the nurse leader was acutely concerned about the quality of the care that was rendered at a time of strict requirements to justify one’s actions professionally.

Two relatively new developments placed additional demands on the municipality’s care services, namely the increase in the numbers of patients with dementia and young patients with multiple sclerosis (MS), who have a need for fully staffed residential care. The provision of care for both of these two patient groups had implications for staff capacity and academic development. One of the nurse leaders conveyed the concerns of the staff as follows:

*It is our wish to excel in care for persons with dementia. We shall excel in what we do… but then we need to have the time for academic development as well. It is quite demanding to work with this patient group and we sometimes feel insufficient in what we have to offer.*

Another one’s views on care for MS patients were:

*We see an increase in patients groups, for example, young persons with multiple sclerosis (MS) who have a need for fully staffed residence quarters because of their illness.*

These health care trends, and the associated inadequate provisions in the budget, left the nurse leader feeling inadequate.

The tasks of the care service were steadily increasing. Several of the informants expressed the need for a physician stationed at specific areas in the care service, ideally in the nursing homes:

*We are constantly having too many patients… we have not been able to follow up with skills. It’s going too fast. What would be ideal was if all the patients’ general practitioners (in Norway, every inhabitant in a municipality is assigned to a general practitioner) were connected to the nursing home, but that may be too early – the change process hasn’t come that far yet.*

It was clear that the personnel operating the care service were overburdened. Current trends of the discharge of increasingly frail, care-needy patients into the municipality’s care service ideally required the increased involvement of general practitioners stationed at the care facilities.

Some nursing homes established short-term stay wards to cope with the steady admission of particularly the care-needy, frail elderly discharged from the hospitals. These units were viewed as extensions of the hospital wards, and several nurse leaders expressed their concerns about these developments as follows:

*We had no choice but to move nurses from other sections of the nursing home to the short-term ward as they couldn’t cope with the slowly increasing pressure in the complex care of this unit. As a result, the short-term ward requires many of our nursing personnel and they need on-the job training at the hospital as well.*

All of these developments indicated that a bottle-neck situation arose in the municipality’s care services who were under strain, both in terms of capacity and skills, to provide the appropriate nursing care.

There were difficulties as well in the recruitment of qualified staff, with one informant summing up the situation as follows:

*We have difficulties with unskilled personnel, which in turn lead to a measure of uncertainty among the nurses. We simply take people off the street because we only have the minimum number of required personnel on duty over weekends, especially during the afternoon shifts and public holidays.*

### Increase in tasks unrelated to nursing leadership

The informants described their average working day as usually hectic, filled with the challenges of reprioritising the one urgent issue over another such that much of what happened was ad-hoc. Their expanded responsibilities under the new reform included contact with the patient’s next of kin, with the latter increasingly aware of their rights as envisioned in the reform. One of the nurse leaders stated:

*I feel as if I am a coordinator in this operation. I’m expected to keep the wheels of this operation turning; to be a connecting link between the wards and the patient’s next of kin. I am nowhere near the patient, but I’m a connecting link between the personnel, the next of kin and the patient*.

These reactions were indicative of the complexities in the balancing act with all the stakeholders. Discussions with the next of kin and the media occupied much of the nurse leader’s day. One expressed it as follows:

*The next of kin have become a powerful group. They think that if they call the local newspaper or the seniors association everything will work out.*

It seems that the next of kin have become more aware of their role of taking part in the care services, and they desired a better service and conditions for their relatives receiving medical care.

The nurse leaders appeared to be committed to the Coordination reform and were motivated to handle the added pressures, provided the necessary budgetary adjustments could be made at municipal level as per the guidelines of the reform. It was therefore not surprising that much time was spent in meetings about finances and reprioritising of tasks. One nurse leader stated it this way:

*There are many discussions about finances, and the professional personnel are faced with many challenges. It is required that I convey the re-prioritising of tasks in a good way.*

Not surprisingly, several nurse leaders felt powerless and inadequate, as one also expressed it:

*I like my job, but sometimes I feel powerless. You are not able to follow up on everything you’re supposed to, my limit has been reached. It is a challenge to work in the nursing and care service.*

The nurse leader’s responsibility for human resource management involved providing guidance to those staff members on sick leave, and interacting with various state entities regarding these matters. One said:

*We are supposed of course to do our job in the best possible way. New tasks are issued to us and sometimes it gets chaotic. I have 300 employees. I do not have the time to follow up on the personnel and our profession adequately. I am also supposed to follow up on those on sick leave who have been away for 3 months or more. The meetings with their respective doctors, the employees on sick leave and the Norwegian Labour and Welfare Administration about how to accommodate these staff in the work place consumes much of my time.*

The above explanation from one of the informants encapsulated the wide range of responsibilities the nurse leader had nowadays. It is then apparent that very little, if any time, was available for the exercise of professional nursing leadership and mentoring.

## Discussion

The preface of the Coordination Reform states that the public health expenditure per capita in Norway is the highest of all OECD countries [10]. Patient queues for specialist health care services have, nevertheless, been steadily increasing for a number of years, with the elderly and chronically ill constituting large segments of the population seeking medical care [10, 21, 25]. Key aims of the new reform include a reduction of hospital bed occupancy rates, the drawing up of “cohesive pathways” for each patient, and greater innovative and collaborative efforts between the two health care sectors. When fully realised, all of these changes may directly impact the administrative load of the nurse leaders who head the frontlines of the municipal health care services. Moreover, the reform provides clear intentions that the municipalities would be allocated a greater share of the health care budget compared to that of the specialist health care services. The main findings of this study reveal rather the opposite (i.e., that the primary health care services, despite having been tasked with more responsibilities, are expected to accomplish “much more with much less”). As a further complication, these increased responsibilities and increased expectations of efficient collaborative interactions across all public service sectors, all within a restricted budgetary framework, are superimposed upon a management model (the NPM) that is not only based on market–oriented principles but also has flattened management structure that is ideally not conducive to transdisciplinary, collaborative interactions [1–4]. Similar responses have been echoed by nurse leaders in other developed countries, whose public health sectors also follow the NPM model [5, 7, 9].

The findings of this study highlight another pertinent issue, namely the extent to which the nurse leader felt that his/her input and recommendations received a fair hearing/response during the deliberations of the municipal executive team. Paradoxically though, the deputy mayor as the de facto CEO of the municipality, was expected to be most familiar with the new judicial, structural and budgetary changes proposed for the municipalities as per the guidelines of the Coordination Reform. It is apparent therefore that the deputy mayors were not adequately prepared and/or underestimated their preparations to implement the new reform. These examples of inadequate communication and sub-par team effort at the municipal executive level may inadvertently foster perceptions that the nursing profession does not receive the recognition it deserves [9, 28]. It might be added in the same breath that health care professionals often feel that they are not identified as key stakeholders either in the development of health policy [9, 28].

As our findings show, nurse leaders are committed health care professionals who are prepared to take on a range of other tasks, even non-nursing related professional tasks. Evidence presented here indicate that the nurse leaders in the Norwegian municipal care services division support the intentions and aims of the Coordination Reform as the spirit of this reform resonates strongly with the oath they took to provide the best care possible for their patients. They expect, however, that their support is reciprocated by all other stakeholders, not least the municipal executive team. The reform proposes rather specific guidelines of more direct and close, follow-up patient care, all of which demands greater resources (staff and budgetary) [11, 12]. Nurse leaders are acutely aware that their management responsibilities are very often different from those of other professions [29], due to the round-the-clock nature of health care service to patients whose lives are dependent on the nursing staff’s ability to see the patient’s every need and execute care accordingly. Lessons could be learned about how organisational changes induced by health care reform have been amicably resolved in other countries [6–8]. For example, a management system termed shared governance, during which the control of nursing practice was delegated to nurses alone, was proposed as a tool to reconcile the conflict between bureaucracy and professional values in achieving organisational goals [30].

Health care reform is here to stay. Nursing professionals are generally not resistant to change, provided they have access to a support system. They will frequently seek collegial support during times of frenetic activity. In their study of occupational stress and organisational commitment among North American nurse leaders, Lee and Henderson [31] likewise found that those leaders with few opportunities to meet with colleagues scored higher on the scale of emotional exhaustion and lower in personal achievement. The results of the present study further show that nurse leaders expressed a willingness to acquire further training and skills. One informant in our study (a leader of a nursing home) was adamant that the nurse leader could benefit greatly from guidance/consultation in daily operations. These requests may not be unreasonable as other studies have shown that continuous guidance was needed in order for the organisation to attain the professional goals it set for itself [32, 12, 33, 34]. Health care regulators may need to consider possible ways to provide guidance and counseling, with telephony and other IT technologies certainly worth considering. Furthermore, given that the Coordination Reform was superimposed on a largely NPM-inspired public service with its flattened two-level management structure, emphasis should be placed on further training opportunities for municipality-based nurse leaders in collaborative skills [24] as these could promote greater delegation of responsibilities in a new way. Moreover, skills in organisational change may be considered as additional desirable qualities amongst leaders and staff.

On a more practical level though, the introduction of the Coordination Reform will bring to the fore the contentious issue of the discharge-readiness of the patients in the specialist health care services [21, 25]. With primary health care services allocated a greater slice of the health care budget under the new reform, the specialist health care services could be expected to demand the speeding up in the discharge rate of patients as a measure to reduce hospital-bed occupancy, which paradoxically, is also a key aim of the reform. The results of the present study show that nurse leaders experienced increased pressure to admit discharged patients whom they feel were clearly not ready for admission into the nursing homes of the municipalities [10, 26], as this places extra demands on resources and municipal nursing staff who often also lack the facilities and skills to administer acute medical care. This could explain the nurse leader’s request for the stationing of general practitioners at the nursing homes. They also would like see improved coordination among all the relevant stakeholders involved in care (the general practitioner’s role, rehabilitation, nursing homes, home care services and specialist health care services) such as to map out meaningful patient pathways as laid out in the reform. The convergence of all of these collaborative endeavors in the best interest of the patient is vital for the confidence of nurse leaders [24–26 29]. Increasing only bed capacity in the nursing homes to tackle the steady stream of frail, care-needy patients discharged from the hospitals does not suffice. The establishment of an administrative office in the municipality that liaises with the specialist health care services and coordinates the placement of discharged patients has lessened some of the administrative workload of the nurse leader to some extent. Much more needs to be done to involve the nursing home staff in the preparation of discharge procedures as they are directly involved in the day-to-day care of the patient [35].

### Strengths and limitations

Our study tapped into the professional knowledge and extensive experience of nurse leaders who are well accustomed to the periodic changes in the Norwegian health care landscape. Their impressions and views are worth considering, particularly since the success of the reform depends on their cooperation and input towards an improved health care system. A limitation of the study is that the experiences of the other stakeholders also affected by the Coordination Reform were not gauged as well.

## Conclusion

The implementation of the Coordination reform has great significance for nursing in general but also for the execution of nursing leadership. Nurse leaders will need the skills, affirmation, support and guidance to function as visible, confident leaders. Findings presented here highlight the immense, complex challenges that nurse leaders face with the introduction of the Coordination reform. As implied in the name of the reform, its success is highly dependent on the efficient collaborative efforts across health care sectors and with external stakeholders (e.g., next of kin and general practitioners). In terms of this new reform, nurse leaders have a responsibility to improve their collaborative interactions with the next of kin such that they acquire the necessary information, training and follow-up regarding the patient. An overriding concern in all of the above is the apparent lack of urgency among key stakeholders (local and national) to find a workable solution regarding an appropriate budgetary framework that could free up the care services to realise their part of the bargain under the new health care reform.

## Additional file

Additional file 1: Questionnaire. (DOCX 11 kb)

## Abbreviations

CEO: Chief executive officer
MS: Multiple sclerosis
NPM: New public management
OECD: Organisation for European Economic Co-operation and Development
