# Supplementary material for: The challenges of primary health care nurse leaders in the wake of New Health Care Reform in Norway
Source: BMC Nurs. 2016 Nov 24;15:66. doi: 10.1186/s12912-016-0187-x (PMC5121988; doi:10.1186/s12912-016-0187-x)
Supplement: Additional file 1: — Questionnaire. (DOCX 11 kb) [file 12912_2016_187_MOESM1_ESM.docx]

**Interview guide**

What are the challenges you face as leader with the introduction of the Coordination Reform?

How is the support you receive from your superiors in the light of the organisational changes proposed by the reform?

What network do you have as leader?

How are your collaborative dealings with the specialist health care services?

Does your work organization have the necessary skills to meet the new objectives as outlined in the reform?
